# Working conditions and occupational well-being of academics in the post-COVID-19 era: a qualitative follow-up study

**DOI:** 10.3389/fpsyg.2026.1927278

**Published:** 2026-09-08

**Authors:** Işıl Karatuna, Sandra Jönsson, Tuija Muhonen

**Affiliations:** 1 Department of School Development and Leadership, Centre for Work Life Studies, Malmö University, Malmö, Sweden; 2 Sociology Department, Lund University, Lund, Sweden

**Keywords:** academics, COVID-19, higher education, job demands and resources, qualitative interview study

## Abstract

The COVID-19 pandemic led to substantial changes in working conditions in academia. This study aimed to explore how academics’ perceptions of job demands, resources and occupational well-being have changed since the COVID-19 pandemic, as well as their expectations and concerns regarding future working practices. To achieve this aim, we conducted a qualitative follow-up study with 16 academics who participated in our previous study during the COVID-19 pandemic (interviews conducted in 2020–2021) and were re-interviewed in 2025. Data were collected through semi-structured interviews and analyzed using qualitative content analysis. The findings indicated that academics continued to experience job demands related to reduced face-to-face communication, hybrid-remote working practices and work overload. In addition, institutional pressure to return to pre-pandemic working conditions was reported as a new job demand. Digital communication options, organizational support and individual digital skills continued to be perceived as resources that supported occupational well-being. Regarding future academic work, some expectations reported during the pandemic period continued into the post-COVID era, particularly flexibility in the choice of workspace, followed by increased use of digital technologies. Some participants also mentioned improved research opportunities and enhanced knowledge production. Concerns regarding reduced face-to-face interaction, management actions and their implications and fully digital education continued over time; however, management-related concerns became broader in the post-COVID era, encompassing issues such as resource constraints, pressure to return to pre-pandemic working conditions and employment insecurity. Some participants also raised new concerns regarding the increasing role of AI in academic work. This study contributes to understanding how academics’ perceptions of working conditions and occupational well-being have evolved since the pandemic. The findings highlight the diverse and sometimes competing perspectives among academics that universities need to consider when organizing and managing future academic work.

## Introduction

The COVID-19 pandemic has fundamentally changed working conditions within academia. With the sudden shift to distance learning, academics had to work from home, teach using digital interfaces and hold online meetings and webinars instead of face-to-face interactions (Marinoni et al., 2020). The transformations experienced during the pandemic did not remain temporary measures. Instead, many of these changes have persisted into the post-COVID era, accelerating the adoption of digital tools and technologies such as remote teaching, Zoom meetings and hybrid forms of work and education (Dewangan et al., 2024; Karatuna et al., 2022). These changes have created several opportunities for academics, such as increased flexibility and more time to concentrate on work (Esteves et al., 2020; Karatuna et al., 2022). However, they have also introduced a range of challenges, including issues related to technology use and acceptance in the work environment, increased administrative burden and concerns regarding equity and engagement in hybrid meetings and lectures (Doğan and Arslan, 2025; Li et al., 2023).

Studies conducted during the pandemic have shown that academics reported reduced motivation, lower work engagement and increased burnout due to increased job demands (Jackman et al., 2022; Karatuna et al., 2022; Taylor and Frechette, 2022). Conversely, those with adequate resources, such as organizational support, faculty training and appropriate home working conditions, were less likely to experience these negative effects (Karatuna et al., 2022; Mäkiniemi et al., 2021; Ueno et al., 2025; van der Ross et al., 2022).

However, relatively little is known about how academics’ perceptions of working conditions and occupational well-being have changed over time. Although a growing body of research has begun to examine academics’ experiences after the COVID-19 pandemic (e.g., Dewangan et al., 2024; Gulliksen et al., 2023; Rahman et al., 2024), less is known about how academics’ perceptions have evolved over time, particularly based on follow-up research involving the same participants. In particular, further research is needed to understand how academics interpret pandemic-related changes in working conditions and how these changes are experienced in the post-COVID era. To address this gap, the present qualitative follow-up study re-interviewed the same participants approximately 5 years after the original study to explore how academics’ perceptions of working conditions and occupational well-being have evolved over time. By following the same participants across the pandemic and post-pandemic periods, this study provides insights into changes and continuities in academics’ experiences, expectations and concerns over time.

### Theoretical framework

The substantial changes in academic working conditions following the COVID-19 pandemic have transformed how academic work is organized and performed, making it important to understand their implications for academics’ occupational well-being. Occupational well-being is a type of work-related well-being defined as the “evaluation of various aspects of one’s job, including affective, motivational, behavioral, cognitive and psychosomatic dimensions” (van Horn et al., 2004, pp. 366–377). Thus, occupational well-being represents a broad evaluation of employees’ work experiences, including how they feel about their work, their motivation and their perceptions of their work situation. Occupational well-being is also related to broader conceptualizations of work-related well-being, which include hedonic aspects, such as positive experiences and satisfaction, as well as eudaimonic aspects, such as meaning and personal growth at work (Fisher, 2010).

One of the most widely used theoretical frameworks for understanding how working conditions influence occupational well-being is the Job Demands-Resources (JD-R) model (Bakker et al., 2023; Demerouti et al., 2001). According to the JD-R model, every occupation involves specific work characteristics that can be categorized as job demands and resources. Job demands refer to physical, psychological, social or organizational aspects of work that require sustained effort and are associated with physiological and psychological costs. In contrast, resources refer to aspects of work that help employees achieve work goals, stimulate personal growth and reduce the negative effects associated with job demands. Within this framework, occupational well-being is shaped by the dynamic interplay between job demands and job resources (Bakker and Demerouti, 2017). While excessive job demands may contribute to strain and impaired well-being, adequate resources can foster motivation, engagement and positive work-related outcomes (Bakker and Demerouti, 2017; Demerouti et al., 2001). Thus, from a JD-R perspective, occupational well-being can be understood as encompassing both hedonic and eudaimonic aspects of work-related well-being. For example, job satisfaction reflects a hedonic form of work-related well-being, connoting pleasure as well as satiation and calmness, whereas work engagement reflects a eudaimonic form of work-related well-being, connoting high activation characterized by enthusiasm, excitement and energy (Bakker et al., 2023).

In higher education contexts, the JD-R model provides a useful framework for understanding how characteristics of academic work may shape academics’ occupational well-being. Previous research has shown that academic work involves a combination of job demands and resources that may influence academics’ work experiences and well-being. Common job demands in academia include high workload, time pressure, administrative responsibilities and multiple role demands related to teaching, research and service activities, whereas job resources include autonomy, social support, organizational support, role clarity, meaningful work and job security (Barkhuizen et al., 2014; Mudrak et al., 2018; Naidoo-Chetty and du Plessis, 2021). Consistent with the JD-R model, research in academic contexts suggests that the balance between these demands and resources plays an important role in influencing academics’ work experiences, motivation and occupational well-being (Bakker et al., 2005; Mudrak et al., 2018).

The COVID-19 pandemic further influenced this balance by introducing new demands and reducing resources in academic work. The rapid transition to online teaching, increased use of digital technologies and blurred boundaries between work and private life created additional challenges for many academics, while reduced face-to-face interaction and social connectedness negatively affected their work experiences and well-being (de Rijk, 2020; Karatuna et al., 2022). Nevertheless, increased flexibility and opportunities for digital work provided additional resources for some academics (Esteves et al., 2020; Karatuna et al., 2022).

Building on this theoretical perspective, the present follow-up study extends our previous research (Karatuna et al., 2022), in which we explored 26 Swedish academics’ experiences of working from home during the COVID-19 pandemic. In that study, using the JD-R model as the theoretical framework, we examined how job demands and resources shaped academics’ experiences and occupational well-being during this period. Overall, the findings indicated that academics experienced lack of face-to-face communication, absence of an academic environment, work overload, and work–home interference as job demands during the pandemic. Supportive resources included online communication possibilities, appropriate working conditions, organizational and social support and individual factors such as experience in online teaching. While many participants reported negative occupational well-being outcomes, those with adequate resources were better able to cope with demanding working conditions. Furthermore, participants expressed expectations for more flexible and digitally supported future working practices, while also raising concerns about the potential loss of in-person interaction and the long-term implications of increasingly digital or hybrid academic work.

### Aim of the study

The present follow-up study aimed to investigate academics’ perceptions of working conditions and occupational well-being in the post-COVID era, as well as their expectations and concerns regarding future academic working practices. Specifically, it examined how academics perceive job demands and resources in the post-COVID era, how changing working conditions have influenced their occupational well-being (e.g., motivation, work engagement, job satisfaction) and whether and how these perceptions have changed compared with the pandemic period. The following research questions were proposed:

1. How do academics perceive their working conditions in the post-COVID era?
2. What are academics’ perceptions of how the changing working conditions have affected their occupational well-being?
3. What are academics’ expectations and concerns related to future academic working practices?

## Materials and methods

Due to the exploratory nature of the research questions, a qualitative descriptive design (Sandelowski, 2000) was used to explore academics’ perspectives.

### Participants and procedure

All 26 participants from our previous study (Karatuna et al., 2022), who had been recruited through purposive and snowball sampling among academics working at various universities in Sweden, were invited via email to participate in this follow-up study. The purpose of the follow-up study was to examine changes and continuities in academics’ experiences and perceptions over time; therefore, the same participants from the original study were invited to participate rather than recruiting a new sample. Therefore, all participants from the original study were invited to participate in the follow-up interviews. Despite considerable efforts, four participants could not be reached and six declined to participate, resulting in a final sample of 16 participants (61.5% response rate). Participants comprised nine senior lecturers, three lecturers, three professors and one researcher. Participants were aged between 38 and 71 years (mean age: 53 years). Of the 16 participants, 5 were women and 11 were men (Table 1). The interviews in our previous study were conducted between October 2020 and January 202 and the follow-up interviews between May and November 2025.

**Table 1:** Participant characteristics.

ID	Position	Gender^1^	Age	Years of experience in academia
P1	Senior lecturer	m	56	27
P2	Senior lecturer	m	59	25
P3	Lecturer	m	50	16
P4	Professor	m	71	40
P5	Senior lecturer	m	38	11
P6	Senior lecturer	f	69	28
P7	Researcher	f	63	18
P8	Senior lecturer	m	47	21
P9	Senior lecturer	m	52	27
P10	Lecturer	f	38	8
P11	Senior lecturer	m	55	18
P12	Senior lecturer	m	55	26
P13	Professor	m	55	23
P14	Lecturer	f	49	9
P15	Professor	m	53	20
P16	Senior lecturer	f	47	23

^1^f = female, m = male.

Data were collected through semi-structured interviews conducted in English by the first author. English was selected as the interview language because it is widely used as a working language in Swedish academia. Furthermore, the first author is not a native Swedish speaker and not all participants were native speakers of Swedish. Thus, English served as a common language between the interviewer and participants. Potential participants were informed in advance that the interviews would be conducted in English and only those who agreed to participate under these conditions were included in the study.

The interviews lasted between 30 and 60 min and were held via Zoom. Participants were interviewed using a semi-structured interview guide which was developed by the researchers based on the research questions. The interview guide included questions on academics’ perceptions of how their working conditions have changed since the end of the pandemic, the challenges and positive aspects of these changes in the post-COVID era, as well as their impact on occupational well-being. It also explored participants’ expectations and concerns regarding future work practices in academia. The interview guide was based on the previous interview guide (Karatuna et al., 2022), which addressed similar topics related to working conditions during the pandemic. The guide used in the follow-up interviews is provided as Supplementary File 1.

The interviews were audio-recorded. All participants provided informed consent for participation and recording.

### Reflexivity statement

The first author (IK) is an associate professor in social psychology specializing in work psychology and organizational behavior. The second author (SJ) is an associate professor specializing in organization theory, working life and health and scientific methodology. The third author (TM) is a professor of work science with a PhD in psychology specializing in organizational and social work environment. Together, the authors have extensive experience in working life research and qualitative research methodologies. This expertise guided the development of the interview guide and the interpretation of participants’ accounts using the JD-R framework.

As this was a follow-up study, the first author had previously interviewed the same participants during the earlier phase of the project. Recognizing the importance of researcher reflexivity and positionality in qualitative research (Berger, 2015), the first author remained aware of the potential influence of this pre-existing relationship throughout the research process. Prior knowledge of participants’ experiences was considered during the analysis to ensure that interpretations reflected the current interview data rather than previous accounts.

Throughout the research process, the authors engaged in ongoing reflexive discussions to critically examine their assumptions, interpretations and analytical decisions, thereby supporting the credibility and trustworthiness of the study (Finlay, 2002).

### Data analysis

The interviews were transcribed verbatim and analyzed using content analysis, a commonly used method for qualitative data analysis that allows both deductive and inductive approaches (Hsieh and Shannon, 2005; Vaismoradi et al., 2013). In this follow-up study, we generated an initial coding frame deductively based on our previous study (Karatuna et al., 2022). However, the coding frame also remained open to the inclusion of any inductively generated codes.

The unit of analysis consisted of the verbatim transcripts focusing on academics’ perceptions of working conditions and occupational well-being in the post-COVID era. The authors individually read the transcripts several times to gain a sense of the whole. They then extracted, condensed and labeled meaning units with codes (Graneheim and Lundman, 2004). After independently coding the transcripts, the authors met on several occasions to compare and discuss their coding. Any differences in coding and interpretation were resolved through discussion until consensus was reached regarding the final coding framework. Subsequently, the codes were compared and those with similar content were grouped into subthemes. No qualitative data analysis software was used and the coding and analysis were conducted manually by the authors. Following the analysis of the follow-up interviews, the subthemes and overarching themes identified through qualitative content analysis were compared with those reported in our previous study (Karatuna et al., 2022) to examine continuities and changes in academics’ perceptions over time. This comparison focused on similarities, differences and shifts in academics’ perceptions between the two time points and did not involve re-analysis of the original interview transcripts.

### Ensuring trustworthiness

To ensure the trustworthiness of the findings, the criteria proposed by Lincoln and Guba (1985) were used: credibility, dependability, confirmability and transferability. Credibility was supported by inviting participants to review their interview transcripts and verify the accuracy of their accounts. Any feedback provided by the participants was taken into account during the analysis. Dependability was strengthened by conducting all interviews using the same interview guide and by involving all authors in the data analysis process. Confirmability was promoted through collaborative analysis among multiple authors and by incorporating direct quotations from the interview transcripts when presenting the findings. Finally, transferability was addressed by providing detailed descriptions of both the participants and the research procedures.

### Ethical considerations

The study was approved by the Swedish Ethical Review Authority (Ref nr. 2025–02267-01). All investigations were carried out in conformity with the ethical standards of the Swedish Research Council (2017). All interviews were conducted after informed consent was provided. Confidentiality was assured and maintained throughout. To protect the anonymity and privacy of the participants, no real names or employer information were used in the study and all data were stored in a secure way preventing access by third parties.

## Results

Using the JD-R model as an analytical framework, the content analysis identified five overarching themes: (1) job demands, (2) job and personal resources, (3) occupational well-being, (4) expectations for future working practices, and (5) concerns for future working practices. Each theme consisted of several subthemes. All themes and subthemes are described in detail below and summarized in Table 2. A comparison of the themes and subthemes identified during the COVID-19 pandemic and in the post-COVID-19 era is presented in Table 3 and the similarities and differences between the two periods are further discussed in the discussion section. Additional information on the themes, subthemes, example codes and the number of participants mentioning each subtheme from our previous study (Karatuna et al., 2022) is provided in Supplementary File 2.

**Table 2:** Themes, subthemes and example codes, including the number of participants mentioning each theme.

Themes	Subthemes	Example codes for each subtheme	Participants mentioning each subtheme (n)
Job demands	Reduced face-to-face communication	Difficulties in having face-to-face contacts with colleagues and students, loss of spontaneous interaction	6
	Adapting to hybrid-remote working	Technical issues, difficulty ensuring equal participation and interaction between in-person and remote participants	6
	Work overload	Increased teaching obligations, administrative burdens	6
	Institutional push for on-campus education	Return to on-campus teaching, require academics to be physically present on campus	5
Job and personal resources	Digital communication options	Flexibility to use digital communication for meetings, teaching and supervision, virtual hangouts with colleagues	16
	Enhanced digital skills	Became more skilled in digital activities, greater sense of comfort and confidence in digital activities	6
	Organizational culture that embraces hybrid-remote work	Institutional support for flexible working practices, normalized on-line participation/ hybrid-remote work	5
	Technical support	IT support to resolve technical issues, IT support and access to technical equipment	3
Occupational well-being	Not affected well-being	Perceived stability in job satisfaction and motivation, not influenced by pandemic-related changes	7
	Positively affected well-being	Greater job satisfaction, better balance between work and private life, enhanced work focus, efficient use of working time, higher concentration	6
	Negatively affected well-being	Disappointment with universities’ eagerness to return to pre-pandemic conditions, disappointment with weakened collegial connections, loneliness	3
Expectations for future working practices in academia	Flexibility in the choice of workspace	Option to choose between working from home and working at university, balance between flexibility and physical presence	10
	Increased use of digital technologies	Use of digital technologies in teaching, collaboration and administrative processes, integrate AI into teaching methods	7
	Improved research opportunities	Research less dependent on external funding, prioritization of research	6
	Enhanced knowledge production	Develop high-quality knowledge, open up universities as creative and inclusive spaces within society	3
Concerns for future working practices in academia	Management actions and their implications	Resource constraints, returning to pre-pandemic conditions, involvement of political actors in the management of academia, loss of academic freedom, employment insecurity	16
	Reduced face-to-face interaction	Not meeting colleagues/students in person, negative impact on the development of new ideas and knowledge	13
	Fully digital education	Teaching all courses through zoom, loss of connection to professional fields in digital education	4
	AI related risks in academic work	Potential threat to traditional academic roles, ineffective integration of AI into teaching and learning	4

Frequencies (n) represent the number of participants who mentioned each subtheme during the interviews.

**Table 3:** Comparison of themes and subthemes identified during the COVID-19 pandemic and in the post-COVID-19.

Themes	Subthemes identified in the post-COVID-19	Subthemes identified during the COVID-19 pandemic
Job demands	Reduced face-to-face communication	Lack of face-to-face communication
	Adapting to hybrid-remote working	Absence of academic environment
	Work overload	Work overload
	Institutional push for on-campus education	Work-home interference
Job and personal resources	Digital communication options	Digital communication options
	Enhanced digital skills	Appropriate working conditions for remote working
	Organizational culture that embraces hybrid-remote work	Organizational and social support
	Technical support	Individuals’ digital skills
Occupational well-being	Not affected well-being	Negatively affected well-being
	Positively affected well-being	Positively affected well-being
	Negatively affected well-being	
Expectations for future working practices in academia	Flexibility in the choice of workspace	Continuation of working online
	Increased use of digital technologies	Flexibility in the choice of workspace
	Improved research opportunities	Strengthened digital capacity/skills
	Enhanced knowledge production	Opportunities for physical meetings
Concerns for future working practices in academia	Management actions and their implications including resource constraints, returning to pre-pandemic working conditions, political influence in academia, reduced academic freedom, employment insecurity	Lack of face-to-face interaction
	Reduced face-to-face interaction	Management actions and their implications including reduced academic freedom, less space at university
	Fully digital education	Fully digital education and hybrid solutions
	AI related risks in academic work	

Within each theme, the identified subthemes are presented in descending order from the most to the least frequently represented to facilitate comparison between the COVID-19 and post-COVID-19 studies.

### Job demands

This theme represents academics’ perceptions of job demands in the post-COVID era and comprised four subthemes: “reduced face-to-face communication,” “adapting to hybrid-remote working,” “work overload,” and “institutional push for on-campus education.”

#### Reduced face-to-face communication

Some participants described increased remote and hybrid working practices as creating challenges for face-to-face interaction. In particular, they highlighted reduced opportunities for spontaneous encounters with colleagues, which they considered important for networking and the spontaneous exchange of ideas. As one participant explained:

“*You want to build a strong network and exchange ideas in the corridors and so on… like spontaneously and these kind of meetings are hard when people don't show up*” (P12)

Some participants also described challenges related to reduced face-to-face interaction with students in hybrid and online teaching. They reported difficulties in maintaining deeper discussions and developing close connections with students when interactions occurred partly or fully online. As one participant described:

“*It's harder to have more deep discussions…You can’t see everyone's facial expressions or reactions…So, you lose some of the sense of close connection.”* (P6)

#### Adapting to hybrid-remote working

Several participants reported difficulties in adapting to hybrid meetings, where some participants were present on-site while others participated remotely. One challenge involved technical problems that disrupted hybrid meetings. As one participant explained:

“*There are huge technical problems, so we spent 10 to 15 minutes just to make sure that the technology works, the connection, the speakers or the camera*.” (P1)

Some participants also described difficulties in ensuring equal participation between in-person and remote participants. They reported that hybrid meetings could become less useful when most colleagues participated remotely and only a few attended in person, making it difficult to involve both groups equally in discussions. As one participant explained:

"*We are quite skeptical about the hybrid mode because there is always a group that is somewhat left out.”* (P9)

#### Work overload

Several participants perceived increased teaching obligations as a job demand. They described teaching-related tasks, such as lesson preparation and dealing with student-related matters, as taking time away from research activities. For example, one participant highlighted the challenges faced by early-career researchers who need to balance teaching responsibilities with research expectations:

“*I see the young researchers spending a lot of time on education and things like that…It takes much time to prepare lessons etc…And at the same time, they need to advance in their research*.” (P7)

Participants also identified administrative responsibilities as a significant job demand. They described increasing administrative workloads as taking time away from research and student-related activities. As one participant explained:

*“We need more time for students, more time for research, less time for administration work and that kind of stuff…I don't know what I'm doing in a normal workday…Sometimes I go back home thinking, I feel like I haven't started working yet even I've been on the work site doing stuff all day.”* (P12)

#### Institutional push for on-campus education

Another job demand identified by participants was institutional pressure to return to pre-pandemic working practices. Some participants described institutional expectations to return to on-campus teaching as challenging, particularly when some activities were perceived as suitable for online formats. One participant described this challenge:

*“One thing I find somewhat disappointing is that university management is very eager to return to on-campus teaching….I believe that certain aspects of teaching, such as thesis supervision, work better via Zoom than in-person meetings*.” (P2)

Similarly, another participant described difficulties with the expectation to conduct student meetings on campus, despite perceiving online meetings as a suitable alternative:

*“I think that is a little bit sad because some student meetings, I think, could easily be done like this. Some students commute quite long distances. Some of our meetings could just be done like this, but we are not allowed to. So, it's quite a step back to how things were.”* (P12)

### Job and personal resources

This theme represents academics’ perceived resources in the post-COVID era and comprised four subthemes: “digital communication options,” “enhanced digital skills,” “organizational culture that embraces hybrid-remote work,” and “technical support.”

#### Digital communication options

All participants described digital communication options as a job resource in the post-COVID era. Platforms such as Zoom and Teams were frequently used for meetings, teaching and supervision. Participants highlighted the convenience and time-saving benefits of these tools, particularly for interactions with colleagues from different campuses or countries. For example, one participant described how digital tools created new opportunities for guest lectures by enabling external speakers to participate remotely while students attended in the classroom:

*One very good opportunity that has shown up is that, if you want guest lecturers, you can bring them into the classroom. It's easy to get people to do that if we can say that you can stream that online. We collect the students in a classroom, we can administrate it a little bit and collect questions and show the presentation online.”* (P9)

In addition to supporting teaching and academic collaboration, digital communication options were also perceived as useful for maintaining social connections among colleagues. For instance, one participant described how they continued to organize online social gatherings and virtual coffee meetings with colleagues, suggesting that such practices enabled continued communication despite physical distance:

*“We have maintained a lot of this digitalized stuff and it's not going away …Every week we continue to have a Zoom breakfast…So we meet everyone who can join, regardless of location.*” (P16)

#### Enhanced digital skills

Some participants identified enhanced digital skills as a personal resource that helped them manage their academic work in the post-COVID era. They described not only increased technical competence in using digital platforms and conducting online meetings, but also greater comfort and confidence when engaging in digital work environments. One participant described how digital skills had developed during the pandemic:

“*People during the pandemic learned both the technique of meeting through Zoom, but also feeling more comfortable being in this kind of meetings.”* (P13)

Participants also highlighted that enhanced digital skills helped them manage online meetings more effectively. One participant explained:

*“I think I learned how to keep meetings short and effective. That is something I learned during the pandemic and I still use that technique.*” (P2)

#### Organizational culture that embraces hybrid-remote work

Some academics perceived an organizational culture that supports hybrid-remote working practices as a supportive resource in adapting to changes in working practices following COVID-19. One participant highlighted how increased flexibility and openness toward hybrid work supported her continuation in academia:

*“I think that they became more open to online hybrid situations and more understanding…they're quite okay and open to having hybrid situations…I think it helped me a lot to be able to continue working in academia.”* (P14)

In addition, some participants described how flexible working practices had become more normalized within academic contexts compared with the pre-pandemic period. They highlighted that working from home and using online alternatives for teaching or supervision had become more common and accepted after the pandemic. One participant explained how working from home had become less questioned:

“*Occasionally I take one of five workdays to work from home…I don't feel guilty about it. Before the pandemic, you had to explain or at least be prepared to have a good excuse for working from home. Today, I don't think anyone would ask…why you didn’t show up yesterday or something like that.”* (P12)

Similarly, another participant described how online teaching and supervision had become accepted alternatives when face-to-face interaction was not possible:

*“One thing that, you know, pandemic changed for us was that now if, for example, I get sick, my students are totally fine with me having a Zoom lecture. So, it's totally accepted that it can be a substitution to some extent for like, you know, if your teacher is sick or like supervision over Zoom can be accepted. So, we mix the teaching more than ever before.”* (P16)

#### Technical support

Some participants highlighted technical support provided by their universities as a job resource in adapting to digital working practices after COVID-19. This support included not only assistance with digital teaching and related activities but also access to the equipment needed for hybrid-remote work. For example, one participant mentioned the support provided by IT services and access to necessary equipment for working from home:

“When it comes to computer equipment, for example, that's been a very supportive view from the IT department. It's no problem to get an extra camera or something like that.” (P9).

Another participant described how technical support staff helped facilitate digital teaching activities:

*“Now the university has employed special staff in the administration to support us with technical solutions…for delivering digital lectures and related activities.”* (P13)

### Occupational well-being

This theme represents academics’ perceptions of their occupational well-being related to changing working conditions in the post-COVID era and comprised three subthemes: “not affected well-being”, “positively affected well-being” and “negatively affected well-being.”

#### Not affected well-being

Some participants reported that their occupational well-being had not been substantially affected by the work-related changes they perceived in the post-COVID era. They described their occupational well-being as similar to before the pandemic. As one participant explained:

“Now, I feel it is just the same as before the pandemic. My job satisfaction is similar to how it was before.” (P1)

Several participants described their well-being as being influenced more by broader aspects of academic work, such as workload, involvement in projects and interactions with students, than by pandemic-related changes. One academic noted:

“*I would not directly attribute my wellbeing to the pandemic-related changes…I think other factors are more important, like your general workload or whether you are engaged in projects and whether students are satisfied with your work*.” (P15)

#### Positively affected well-being

Some participants perceived that changes in working conditions in the post-COVID era had positively influenced their occupational well-being. They described increased flexibility and the continued use of digital communication options as improving their working conditions. One participant highlighted how reduced commuting enabled more efficient use of working time:

“*I can easily stay at home…I have deadlines, so staying home actually makes more sense. I do this to get extra time to prepare and then I can use the hour I would normally spend commuting.*” (P9)

Some participants also described flexible working conditions as contributing to their quality of life and enabling a better balance between work and private life. One participant explained:

“I think it increases your quality of life. I can still pick up my son from the daycare while, having this meeting with you online. So, it just makes things easier, especially with our living conditions and the fact that not everybody is living so close to their work.” (P14)

Some participants also described working from home as supporting concentration and allowing them to focus more on academic tasks. As one participant described:

“*Now I have more time to work deeper on my own. You don't have those discussions about how anyone feels or their family situation or whatever you might have been doing when you met more often on campus.*” (P6)

#### Negatively affected well-being

A smaller group of participants perceived that changes in working conditions in the post-COVID era had negatively influenced their occupational well-being. They described reduced spontaneous interactions and weaker connections with colleagues as factors contributing to lower well-being. As one participant mentioned:

“For my working*…*I think it has been more negative than positive in that way. We don't have that*…*we are not so close to each other anymore and it has been more like we are isolated from each other. You don't get that spontaneous connection in the corridor or in the round of a break or so*…*It feels sad.” (P3)

Similarly, reduced face-to-face communication was described as limiting opportunities for informal interactions and spontaneous exchange. One participant described disappointment that fewer colleagues were present at the university, which made it more difficult to build connections and exchange ideas:

*“I'm a little disappointed with people who are not showing up at the office…Why are only three of us out of 60 here at lunchtime?…You want to build a strong network around you and exchange ideas spontaneously in the corridors, but these kinds of meetings are difficult when people don’t show up.”* (P10)

### Expectations for future working practices in academia

This theme represents academics’ expectations for future academic working practices and comprised four subthemes: “flexibility in the choice of workspace,” “improved research opportunities,” “increased use of digital technologies,” and “enhanced knowledge production.”

#### Flexibility in the choice of workspace

Many participants reported that they would like to retain flexibility in their choice of workspace in the future. They described flexibility as important for making academic work better suited to individual circumstances. For example, one participant highlighted the importance of flexibility when academic positions were located far from where people lived:

*“My expectation of an academia…an academia that would put people first, not jobs first…allowing for fluidity and flexibility…I mean, it would be very nice to get a job at a university close to my home. But the jobs are limited and often located in different places. So, it becomes even more important for work to be accessible.”* (P14)

Another expectation regarding future working practices was that universities should adopt more flexible approaches that consider individual needs and roles. One participant highlighted the need for approaches to workspace flexibility that consider different roles and responsibilities:

“*As a head of department, it wouldn't be possible to work this much from home because you need to be where lecturers, professors and students are…I'm arguing for a kind of nuanced approach that is based on individual needs.*” (P11)

Although some participants valued flexibility, others emphasized the importance of maintaining a certain level of on-campus presence. They suggested that future working practices should combine flexible working arrangements with regular opportunities for face-to-face interaction on campus. As one participant noted:

*“I think the academia should establish something similar to what many workplaces have defined…that you need to be there three days a week. I think that could be a good level for everyone.*” (P9)

#### Increased use of digital technologies

Another expectation for future academic work was the increased use of digital technologies in teaching, collaboration and administrative processes. Participants highlighted the potential of digital tools to expand access to teaching activities across institutions. For example, one participant described how Zoom-based teaching enabled the development of a national master’s course:

“*I was involved in a project where we tried to organize a national master’s course. This was made possible by technologies such as Zoom…But we are still too tied to the local interests of our universities and therefore can’t fully see the possibilities that exist at the national level…We can create new digital courses that benefit everyone.*” (P2)

Participants also highlighted the need to rethink teaching and assessment practices in light of digital developments, including artificial intelligence (AI). One participant questioned the continued reliance on traditional essay-based assignments and suggested exploring alternative approaches that incorporate digital tools, including AI:

*“I think the time of writing essays and submitting them is over…we should move beyond that. Maybe we could work in groups to find answers to questions using any tools, including AI, and then discuss the findings in groups and seminars.”* (P5)

#### Improved research opportunities

Another expectation for future academic work was better opportunities for conducting research. Participants highlighted the importance of having sufficient time and supportive conditions for academic research. One participant described how external funding requirements could limit the time available for research activities:

“*For me, the system is not ideal because the only thing people do is apply for funding…leaving no time for actual research. An ideal position would be research built into a position rather than externally funded.”* (P16)

Participants also emphasized the importance of a research environment that encourages engagement and collaboration. One participant described the need for a more dynamic research environment that encourages academics to engage in research activities:

“*Academics have dedicated time for research but not many colleagues use that time for research. My dream scenario of the academia would be an environment for research that is so dynamic and interesting that people would choose to prioritize it.*” (P13)

#### Enhanced knowledge production

Some participants highlighted the importance of universities maintaining their role as trustworthy knowledge-producing institutions. One participant emphasized the importance of universities continuing to produce reliable knowledge and maintain public trust in academic research:

“*I think academia is challenged as a knowledge producer…A lot of research is performed by companies and that is not always publicly available…Academia should find ways to be robust and trustworthy knowledge producer that propagate scientific methods.”* (P15)

Another participant expressed expectations that universities should remain open spaces for interaction and idea development:

*“In the dream scenario, I would say that the interaction with the students, opening up universities, being a space where people could develop their thoughts, experiments…challenging established norms and values and more creative space within the society because these spaces or arenas are quite few nowadays…”* (P13)

### Concerns for future working practices in academia

This theme describes academics’ concerns about future working practices in academia and comprised four subthemes: “management actions and their implications,” “reduced face-to-face interaction,” “fully digital education,” and “AI related risks in academic work.”

#### Management actions and their implications

Participants raised concerns about university management actions and their implications for future working practices in academia. These included resource constraints, the return to pre-pandemic working practices, perceived political involvement in academia, academic freedom and employment insecurity. For example, one concern related to reduced resources and their possible effects on academic programs and staff, particularly in the social sciences:

“*My concerns are more related to the fact that we are saving money, the cuts to social sciences, for example resources to social sciences and humanities are shrinking… programs are being cut down, courses are being taken out, people have to find other kinds of work.”* (P16)

Participants also expressed concerns about perceived political involvement in academia and its implications for academics’ freedom to express their views. These concerns were reflected in fears of self-censorship:

*“I think that we, academics, are extremely bad to stand up for ourself and it's strange how little we are protesting if there is political involvement in what we are doing and or what we are saying or should be saying and so forth… Lecturers are afraid of what they can say and not say in the classroom. Increasing political involvement in academia might lead to self-censorship among us.”* (P12)

Relatedly, some participants described increasing monitoring and control within universities as potentially limiting academic freedom and autonomy:

*“Also there is this fact that your performance is monitored more and more … this gives you less freedom in your academia. And that’s a part of the working conditions. … you see more and more control from vice-chancellors.”* (P4)

Concerns were also raised about institutional efforts to return to more traditional on-campus working practices. Some participants perceived these changes as reducing the flexibility gained during the pandemic period:

“*Some universities have become quite old school again…returning to a strictly on-campus model. I think this would reduce accessibility and limit opportunities for a wider population to be part of academia*.” (P14)

Finally, some participants expressed concerns about employment insecurity in the future of academic work. One participant described the lack of stable positions, particularly for early career researchers:

“*Early career researchers don't get steady positions…They must leave after two years*.” (P13)

#### Reduced face-to-face interaction

Another concern regarding future working practices in academia was reduced face-to-face interaction among colleagues. Some participants emphasized the importance of opportunities for in-person interaction and collaboration within university environments. As one participant explained:

“*The university just becomes a work hotel, where people are not really there. It’s difficult to get them together, and then the space can’t serve as a multidisciplinary meeting place to create new ideas and collaborations… So, we have to work with this kind of arena to have people and to get them together because they're not always meeting in the coffee room anymore.*” (P9)

In addition, some participants expressed concerns that increased working from home could reduce spontaneous interactions among academics. They described these informal interactions as important for collaboration and knowledge development. As one participant explained:

“*The worst-case scenario is that everyone is working single without connection to other lecturers… working together is important for developing knowledge… you need to meet each other*.” (P6)

#### Fully digital education

Some participants raised concerns about a possible shift toward fully online education in future academic working practices. Although digital tools were generally viewed as useful, participants emphasized the importance of face-to-face teaching and interaction. For example, one participant highlighted the value of classroom-based teaching:

“*We have seen the importance of actually meeting in the classroom and discussing. We see that the results during the pandemic were not entirely as good as they would be in ordinary teaching.*” (P9)

Participants also emphasized that students’ connection to professional contexts was an important part of learning. One participant described concerns that fully online education could weaken this connection:

“*I think it's very important to know about the field where you should be working…So those students that don’t have close connection to their working fields would not learn enough.”* (P6)

#### AI related risks in academic work

Some participants expressed concerns regarding the increasing role of AI in academic work, including research and teaching. They particularly raised concerns about the use of AI-generated articles in academic publishing. One participant stated:

“*It becomes quite pointless if we have journals publishing thousands and thousands of articles that have been produced by AI instead of people. There is no point doing research anymore.”* (P1)

Although AI was also recognized as a useful tool for supporting learning, some participants expressed concerns that differences in how students use AI could contribute to inequalities in learning outcomes. One participant described how AI use could benefit some students while others may use it in ways that do not support learning to the same extent.

“*The good students use AI in a very good way to add on their knowledge but the not so good students they use AI to find shortcuts…it tends to actually divide them a little bit more.”* (P9)

Some participants expressed concerns that academic practices have not yet fully adapted to AI. For example, one participant described the challenges of adapting teaching practices to AI and the need to develop strategies for working with AI rather than against it:

“*We have this AI that is taking over now…and we don’t know how much the students are actually learning…And we are trying to put out fires and implement changes to accommodate AI, but we are not really rolling with it… we need to find strategies to work with AI, not against it, because it’s here to stay.*” (P5)

## Discussion

The findings of our study indicated that, approximately 5 years after the initial pandemic-period study (Karatuna et al., 2022), academics’ perceptions of working conditions and occupational well-being reflected both changes and continuities. In both periods, participants perceived reduced face-to-face communication as a job demand and emphasized the importance of in-person academic interactions. During the pandemic, this was particularly related to the absence of academic environments, opportunities for networking and informal discussions with colleagues, while follow-up interviews highlighted ongoing challenges related to spontaneous interactions within hybrid-remote working practices. This finding is consistent with previous research highlighting that academics may experience difficulties in maintaining spontaneous interactions and informal exchanges in hybrid-remote working contexts (Batisse et al., 2026; Tinnerholm Ljungberg et al., 2025).

Another job demand identified across the two periods was work overload. During the pandemic, work overload mainly reflected the demands of the rapid transition to online education, which required academics to learn new teaching techniques and adapt their courses to digital formats within a very short period. In contrast, in the post-COVID era, work overload no longer reflected the transition itself but was more often associated with increased teaching obligations and administrative responsibilities.

In addition, adapting to hybrid-remote working practices represented a more specific job demand in the post-COVID era. While challenges related to digital adaptation were also present during the pandemic period, follow-up interviews highlighted ongoing difficulties related to technical issues and ensuring equal participation and interaction between in-person and remote participants (Li et al., 2023). Participants also described institutional efforts to return to pre-pandemic working practices (Gutman et al., 2024) as an additional job demand in the post-pandemic academic environment.

Our findings showed that academics reported similar personal and organizational resources when adapting to changing working practices in the post-COVID era. These included digital communication options, digital skills and organizational approaches that support hybrid-remote working. This is consistent with previous research showing that virtual communication can support social interaction and connectedness during periods of reduced face-to-face communication (Sjølie et al., 2020; Uusiautti et al., 2024). Previous studies have also highlighted the role of digital competencies and organizational support in adapting to digitally supported working practices (Dinu et al., 2021; Gulliksen et al., 2023).

During the pandemic, many academics reported negative effects of high job demands on their occupational well-being, while individual and organizational resources were perceived as supportive in managing these challenges. In the post-COVID era, experiences became more diverse, with fewer participants reporting negative effects related to changing working conditions, while others described positive effects or no substantial changes in their well-being. These differences appeared to be related to the combination of job and personal resources available to participants. For example, organizational support for flexibility and hybrid-remote working practices was perceived by some participants as a supportive resource that improved work-life balance and enhanced occupational well-being. Similarly, Tinnerholm Ljungberg et al. (2025) reported that flexibility and hybrid working practices increased employee satisfaction by improving work-life balance and reducing commuting time. However, our findings also indicate that the same working practices were experienced differently across participants, with some perceiving them as resources that supported occupational well-being, while others associated them with challenges that negatively affected their work experiences and occupational well-being. For instance, digital communication tools and hybrid-remote working practices were perceived by some participants as resources that increased flexibility and supported communication, whereas others associated these same practices with reduced spontaneous communication and technical difficulties. Similar findings have been reported by Jaß et al. (2024), who showed that hybrid work can function either as a job demand or a resource, as reduced social interaction may be experienced negatively by some employees, whereas others benefit from increased autonomy and opportunities for focused work.

Overall, our findings are consistent with the JD-R model by suggesting that job demands were often associated with negative occupational well-being experiences, whereas job and personal resources were perceived as supporting academics’ adaptation to changing working conditions and contributing to more positive occupational well-being experiences (Demerouti et al., 2001). Our findings also highlight the dynamic nature of job demands and resources in post-pandemic academic work, showing that the same working practices may function as either demands or resources depending on individual circumstances and organizational contexts (Jaß et al., 2024; Marozva and Pelser, 2025). Thus, job demands and resources should be understood as context-dependent experiences rather than fixed categories (Bakker and Demerouti, 2017).

While the JD-R model helped explain how changing working conditions were perceived as job demands or resources and how they influenced occupational well-being, participants’ expectations and concerns provided insights into the possible directions of future academic work. These expectations and concerns were considered as complementary findings rather than as additional components of the JD-R model. During the pandemic, these expectations and concerns mainly centered around whether newly adopted practices, such as hybrid working, working from home and fully digital education, would remain in academia after COVID-19. In the follow-up interviews, however, hybrid-remote working practices were increasingly perceived as an ongoing part of academic work rather than temporary changes, consistent with research showing that hybrid-remote working has become increasingly normalized in higher education after COVID-19 (Gutman et al., 2024). While some participants valued flexibility, digital technologies and new opportunities for collaboration, they also expressed concerns regarding reduced face-to-face interaction, fully digital education and its implications for teaching practices. Similar concerns have been discussed in previous research, particularly regarding the opportunities and limitations of digital teaching and the importance of maintaining face-to-face interaction (Eringfeld, 2021; Hietanen and Svedholm-Häkkinen, 2023; Li et al., 2023).

Concerns regarding management actions and their implications were particularly emphasized by participants. These concerns reflected broader structural challenges in academic work, such as resource constraints, academic freedom and employment insecurity, which have also been discussed in previous research (Alibašić et al., 2024; Pineda and Salazar Morales, 2024). Moreover, some participants raised concerns about the increasing role of AI in academic work. These concerns extended beyond the adoption of new technologies and involved broader questions regarding changing academic roles, academic work practices and educational processes in higher education. Such concerns are consistent with recent discussions on the implications of generative AI for higher education (Lee et al., 2024; Mohammadi et al., 2026; Watermeyer et al., 2024).

Overall, the findings of this study indicate that academics hold diverse and sometimes competing perspectives regarding post-pandemic academic work. These differences were reflected in how changing working practices were experienced as job demands or resources, as well as in academics’ expectations and concerns regarding future working practices. Rather than suggesting a single model for future academic work, our findings highlight the need for universities to consider these diverse perspectives when organizing working practices. Future academic work should not be based on either a complete return to pre-pandemic practices or a fully digital model of academic work, but should combine the benefits of flexibility, digital opportunities and face-to-face interaction.

Importantly, universities should recognize that specific working practices may not function as demands or resources in the same way for all academics. Therefore, future approaches should consider academics’ diverse needs and working contexts when addressing changing working conditions. This includes providing support for academics’ adaptation to changing working conditions through digital skill development opportunities, adequate technical support and organizational practices that enable hybrid-remote working, while also ensuring opportunities for face-to-face academic interaction. At the same time, broader structural challenges related to academic work, including resource constraints, insecure employment and academic freedom, should be considered when developing future work practices. Finally, the increasing role of digital technologies, including AI, represents an area requiring further consideration. Although concerns regarding AI and digital education were raised by a limited number of participants, these perspectives highlight potential challenges associated with future academic practices. However, they should be interpreted as emerging perspectives rather than representing the views of academics more broadly.

The current study had several limitations. Although the follow-up design provided valuable insights into changes in academics’ experiences over time, only 16 of the 26 participants from the initial study participated in the follow-up interviews. However, retaining participants over extended periods is a common challenge in follow-up studies (Gustavson et al., 2012). Moreover, attrition may have introduced some bias, as the follow-up sample included relatively few early-career academics and a lower proportion of female academics. As the participants were predominantly more senior academics, the experiences of early-career academics, who may face greater employment insecurity and precarious working conditions (McKenzie, 2021), may be underrepresented. Therefore, concerns related to job insecurity and employment conditions identified in this study may not fully capture the experiences of academics across different career stages. Similarly, the lower representation of female academics may have limited the extent to which gender-related differences in experiences of academic work were reflected in the findings (Minello et al., 2021). Future research should further examine post-pandemic working practices across different career stages and gender groups.

Moreover, the findings were based on academics working in Swedish higher education institutions and national differences in higher education systems, employment conditions and organizational structures may influence experiences of changing working practices. For example, characteristics of the Swedish higher education context, including institutional autonomy and governance structures, should be considered when interpreting the transferability of the findings (Karran et al., 2023). Future research should further examine post-pandemic academic working practices across different institutional and national settings. Despite these limitations, our study contributes to the emerging literature on post-pandemic working conditions in academia by examining how academics’ experiences, expectations and concerns changed from the pandemic period to the post-COVID era and by highlighting changes in job demands, resources and occupational well-being over time.

## Data Availability

The datasets presented in this article are not readily available because the interview datasets are subject to ethical and confidentiality restrictions and are therefore not publicly available. The manuscript provides a comprehensive summary of the data and the findings. Further inquiries can be directed to the corresponding author. Requests to access the datasets should be directed to isil.karatuna@mau.se.
