# Supplementary material for: Working conditions and occupational well-being of academics in the post-COVID-19 era: a qualitative follow-up study
Source: Front Psychol. 2026 Sep 8;17:1927278. doi: 10.3389/fpsyg.2026.1927278 (PMC13593505; doi:10.3389/fpsyg.2026.1927278)
Supplement: Supplementary file 1 [file Data_Sheet_1.PDF]

Interview guide

1. Background information

- Can you tell me about your current occupational position and your main tasks?

- How long have you worked in academia?

- Your age?

2. Working conditions during the pandemic

- Was it mandatory for you to work from home during the Covid-19 pandemic? If yes, for how long did you work from home? If no, how often were you at university?

- Did you also work from home before the Covid-19 pandemic? If so, how many days a week?

- What were the biggest changes in working methods/approaches during the Covid-19 pandemic? (e.g., changes in your workload, how you plan your work, your teaching methods, your research activities, your working conditions, sufficient space/materials, how you communicate with others (colleagues, superiors, students)

- Were there any positive aspects of working from home? If so, which ones? In what way?

- Were there any challenges with working from home? If so, which ones? In what way?

3. Current working conditions

- How have your working conditions changed since the pandemic ended?

- What do you think about these changes? Have these changes created any challenges for you? Have these changes brought any positive aspects or benefits?

- Which of the changes introduced during the pandemic have remained stable in your work environment?

- Have any of these changes disappeared over time? If so, which ones and why do you think they have disappeared?

4. Occupational well-being

- How have these changes affected your work well-being (e.g., job satisfaction, work engagement, motivation)?

- Do you feel that your well-being at work has improved or worsened since the pandemic ended? In what way?

5. Concerns and expectations

- What expectations do you have for future working conditions and work environment within academia?

- What concerns do you have for future working conditions and work environment within academia?

- If you were to decide on the future way of working in academia, how would you design it? Tell me about your dream scenario. Why is this a dream scenario for you?

- What would be your worst-case scenario about future work patterns in academia? Why is this a worst-case scenario for you?

6. Other comments

- Is there anything else you would like to add about your experiences with working conditions and work well-being in the post-pandemic era?

Thanks for the conversation!
