# Supplementary material for: Working conditions and occupational well-being of academics in the post-COVID-19 era: a qualitative follow-up study
Source: Front Psychol. 2026 Sep 8;17:1927278. doi: 10.3389/fpsyg.2026.1927278 (PMC13593505; doi:10.3389/fpsyg.2026.1927278)
Supplement: Supplementary file 2 [file Data_Sheet_2.PDF]

Table 2: Themes categories and example codes, including the number of participants mentioning each theme

Themes	Subthemes	Example codes for each subtheme	Number of participants mentioning each subtheme (n)
Job demands	Lack of face-to-face communication	Lack of face-to-face communication with students/ colleagues, communication barriers in online teaching	26
	Absence of academic environment	Miss discussions with colleagues in person, lack of networking in conferences/seminars	19
	Work overload	Back-to-back zoom meetings, learn new teaching techniques in a short time	17
	Work-home interference	Lack of appropriate working space, blurred boundaries between work and private life	7
Job and personal resources	Online communication options	Easy attendance to different work-related activities, virtual hangouts with colleagues	19
	Appropriate working conditions	Having adequate space at home or outside, good internet connection	18
	Organizational and social support	Support from IT department/colleagues/manager, having someone to talk to at home	15
	Individual factors	Being experienced in online teaching, good phase in research activities	8
Occupational well-being	Negative outcomes	Decreased motivation, feeling tired of long zoom days, feeling lonely, decreased work engagement	23
	Positive outcomes	Healthier lifestyle, efficient working, developed digital skills	11
Expectations for future working practices in academia	Continuation of working online	Continue workplace meetings by zoom, partial digitalisation of teaching	21
	Flexibility in the choice of work space	Option to choose between working from home and working at university, increased tolerance of not being in place	11
	Strengthened digital capacity/skills	Good digital capacity, universities that enhance home-based work environment	7
	Opportunities for physical meetings	Smaller offices for concentrated collaborative work, coffee rooms where students and teachers come together	6
Concerns for future working practices in academia	Lack of face-to-face interaction	Not meeting colleagues/students in person, falling apart of social groups	25
	Management actions and economic implications	Less academic freedom, less space at university	9
	Pure digital education and hybrid solutions	Teaching all courses through zoom, pure digital meetings better than hybrid ones	7

Note. Frequencies (n) represent the number of participants who mentioned each subtheme during the interviews.
